# A mixed-methods feasibility study of an intervention to improve men’s mental health and wellbeing during their transition to fatherhood

**DOI:** 10.1186/s12889-021-11870-x

**Published:** 2021-10-08

**Authors:** Sharin Baldwin, Mary Malone, Trevor Murrells, Jane Sandall, Debra Bick

**Affiliations:** 1 Warwick Clinical Trials Unit, University of Warwick, Warwick, UK; 2 Learning and Organisational Development, London North West University Healthcare Trust, London, UK; 3 Oxford School of Nursing and Midwifery, Oxford Brookes University, Oxford, UK; 4 Florence Nightingale Faculty of Nursing, Midwifery & Palliative Care, King’s College London, London, UK; 5 Department of Women and Children’s Health, School of Life Course Science, Faculty of Life Sciences & Medicine, King’s College London, London, UK

## Abstract

**Background:**

Many health visiting services in England use the Promotional Guide system with mothers and fathers, an intervention to support their transition to parenthood, but there is little known about its use and effectiveness, especially with fathers. The aim of this study was to test the feasibility and acceptability of the Promotional Guide system with first-time fathers and pilot potential outcome measures to assess their mental health and wellbeing.

**Methods:**

A mixed methods prospective observational cohort study. Expectant first-time fathers were recruited from four London (UK) local authority boroughs. Data were collected through online pre and post intervention questionnaires, and semi-structured telephone interviews. Quantitative data were analysed using descriptive statistics and qualitative data were analysed using framework analysis.

**Results:**

Eighty-six fathers were interested in participating; 7 did not meet inclusion criteria and 79 were invited to complete the baseline questionnaire. Questionnaires completed by 45 men at both timepoints were included in the final analysis. Mean and standard deviations were calculated for all outcomes, showing a slight deterioration in the scores across all measures in the postnatal period compared to the antenatal. Ten of these men were also interviewed. Six major categories were identified: 1) Experience of health visitor contact, 2) Experience of Promotional Guides, 3) Experience of perinatal health services, 4) Experience of fatherhood, 5) Fathers’ mental health and wellbeing, and 6) Experience of the research process.

While antenatal and postnatal outcomes were collected from 45 first-time fathers, none had received the intervention in its entirety. This study identified major gaps in the implementation of the Promotional Guide system with fathers.

**Conclusion:**

This study assessed recruitment of first-time fathers, time to complete recruitment, and retention rates and identified outcome measures that could be used in a future definitive study. While it wasn’t possible to examine the potential changes following the use of the Promotional Guide system, the study reported on the changes in the fathers’ ‘states’ in the antenatal and postnatal period. It provided a narrative on whether first-time fathers found it acceptable to be asked about their mental health and wellbeing, highlighted their specific needs during their transition to fatherhood, and how they wanted to be supported. It also identified barriers to implementation of the Promotional Guide system by health visitors, which need to be addressed prior to any future research into this intervention. These findings have a number of implications for researchers, health professionals, health service managers, commissioners, policy makers and parents.

**Supplementary Information:**

The online version contains supplementary material available at 10.1186/s12889-021-11870-x.

## Background

Fathers’ mental health during the perinatal period has received more attention in recent years, with research suggesting depression experienced by up to 10% [1], and anxiety by up to 18% [2] of fathers in the antenatal and postnatal periods. The period from an infant’s conception to the age of two is a crucial time for child development, and experiences during this time are likely to influence the rest of the child’s life [3]. Similar to the impacts of maternal depression, mental health problems in fathers are associated with cognitive, emotional, social and behavioural problems in children [4–6]. Poor mental health in fathers also means that they may not be able to adequately support their partners in the perinatal period [7, 8]. Support for new fathers and addressing their mental health needs during this period is therefore important for the wellbeing of the whole family.

Health visitors, who are specialist public health nurses in the United Kingdom (UK) lead and deliver the national Healthy Child Programme (HCP) [9], working with parents from pregnancy until their child’s fifth birthday. Many health visiting services in England use an intervention known as the Promotional Guide system with mothers and fathers, to support their transition to parenthood [10]. The Promotional Guide system is based on the Family Partnership Model, designed to promote early child development and the transition to parenthood, and consists of an antenatal guide used during 34–36 weeks of pregnancy and a postnatal guide used around 6–8 weeks after the baby’s birth [10]. Although the Promotional Guides are designed to be used with mothers and fathers, the HCP in England recommends its use with women during the antenatal and postnatal health visitor contact [11]. It is also recommended in the WAVE Trust (Worldwide Alternatives to ViolencE, an international educational charity) report ‘Conception to age 2 – the age of opportunity’, produced to guide national and local decision-makers and commissioners in how to reduce causes of disadvantage at the earliest and most effective point in life [3].

The Promotional Guides are used face-to-face with mothers and fathers together, by health visitors trained in their use, taking approximately 60 min to complete the guide. They include questions based around five core themes:

- Health, wellbeing and development of baby, mother and father
- Couple relationship
- Family and social support
- Parent-infant care and interaction
- Developmental tasks of early parenthood and infancy

The key components of the Promotional Guide System are: Antenatal and Postnatal face-to-face contacts with mother and father, Antenatal and Postnatal Promotional Guides, Antenatal and Postnatal Topic Cards, Strengths and Needs Assessment, Staff training (skills) and supervision (quality), and Partnership approach between parents and professionals [12, 13].

Although the guides focus on a family’s transition to parenthood and not specifically on paternal mental health, the guidance offers the potential to support fathers’ mental health and wellbeing during this period through the following processes:

1. The intervention enables fathers to discuss their experiences of fatherhood at two points in the perinatal period (antenatal and postnatal) and identify any difficulties they may face.
2. The guides focus on strengths and through self-analysis could help identify ways of addressing any difficulties.
3. The intervention could have a therapeutic effect on fathers, as it has reported among mothers [14].
4. The intervention may enable fathers to identify their need for additional mental health support requiring referrals.

**Fig. 1:**
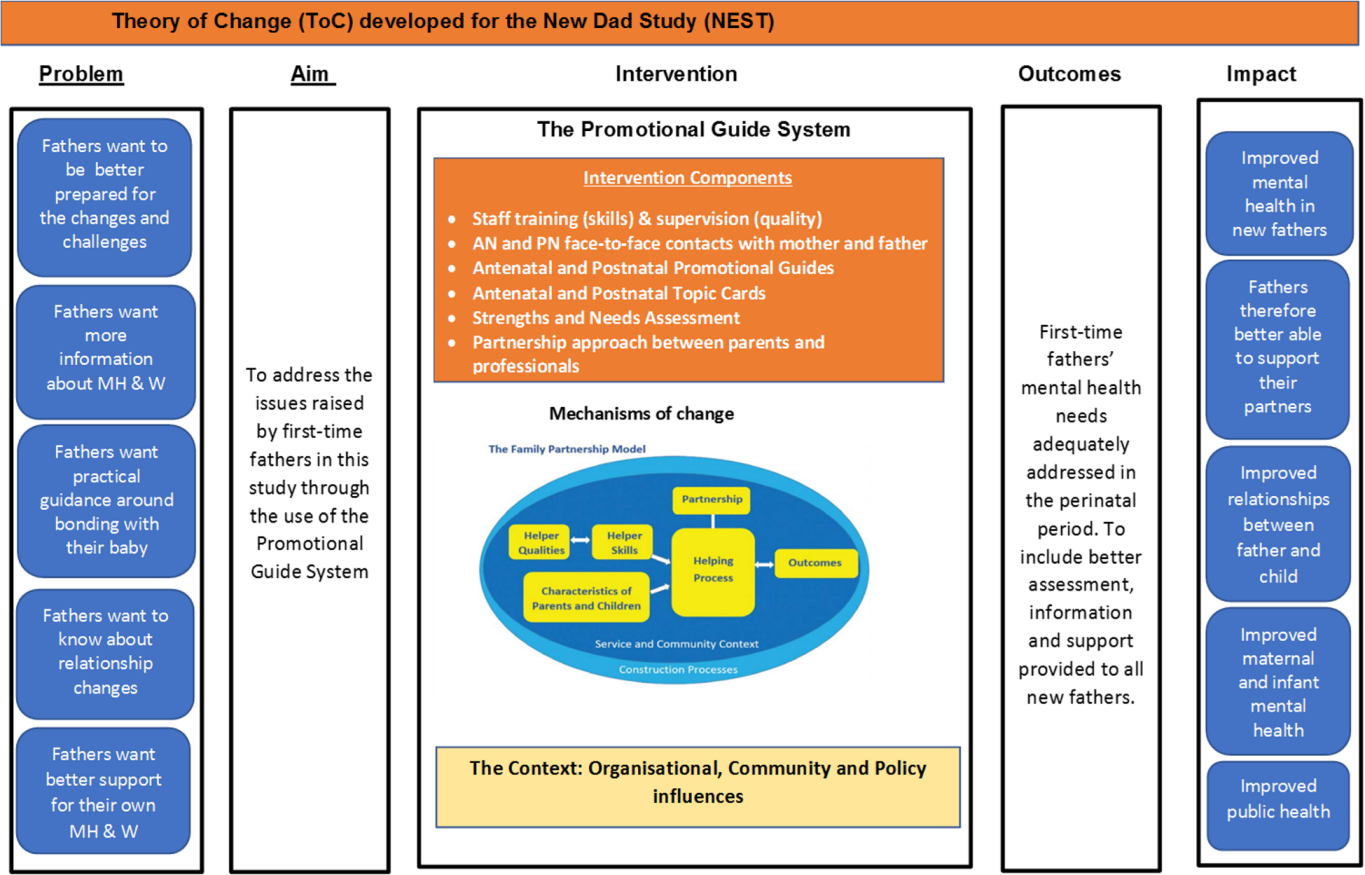
Theory of Change (ToC) developed for the New dad Study (NEST)

**Fig. 2:**
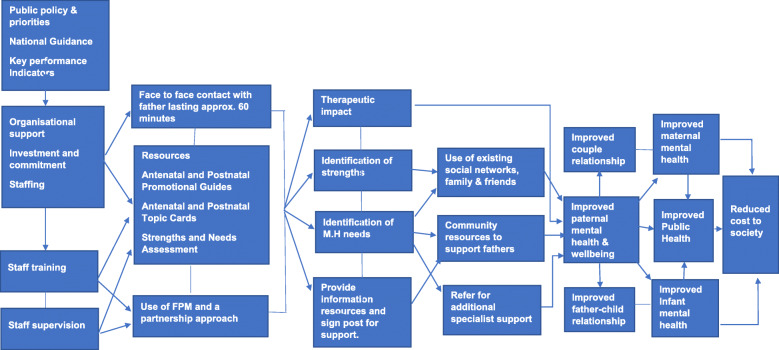
Logic model for improving first-time fathers’ mental health and wellbeing using the Promotional Guide System

A theory of change and logic model (Figs. 1 & 2) was developed to identify the mechanism by which the Promotional Guide system might improve first-time fathers’ mental health and wellbeing during their transition to fatherhood.

The first version of the Promotional Guides was produced in 2000 and referred to as Promotional Interviews, for the European Early Promotion Project (EEPP) [15], which was evaluated across five European countries - the UK, Finland, Greece, Serbia and Cyprus. The evaluation involving 824 families by the programme developers showed some positive outcomes such as parents’ greater satisfaction with and perceived helpfulness of the healthcare professionals trained in the promotional methods; the practicalities of the service in the intervention group were perceived more favourably than the usual services by women in Cyprus, Greece and the UK; and women in all five countries stated that their healthcare professionals made them feel more positive about themselves [16–18].

Following the inclusion of the Promotional Interviews in the HCP [19], the first UK study examining its implementation reported that the guides were rated highly by providers (health visitor) and recipients (women) [14]. This was a mixed-methods evaluation which aimed to assess the level of implementation and stakeholder perceptions, using an online survey with trained health visitors and interviews with key stakeholders (health visitors/ managers, and women receiving the intervention). Questionnaires were completed by 47 health visitors (46% of those invited), only 6 (13%) of whom were using the Antenatal Promotional Guides with all women and 13 (29%) the Postnatal Promotional Guide. Although a very small study carried out in one National Health Service (NHS) setting in London, where implementation of the intervention was low, qualitative findings from interviews with seven women suggested that they were appreciative of the listening, support and guidance provided by the health visitors through the Promotional Guide contacts [14]. Fathers were not included.

Despite the lack of robust evidence in the UK setting, the Promotional Guides are now used by health visitors in eighty-five NHS trusts across England (as of 2018, when the study was undertaken). While a number of service audits have taken place, no primary research was identified (as searched on UKCTG and ClinicalTrials.gov website at the start of this study in June 2018), and questions relating to the level of engagement and acceptability especially by fathers (to include fathers from diverse ethnic and disadvantaged backgrounds) remain unknown, as well as whether the intervention is likely to be of benefit.

In recent years another licensed parenting programme, the Family Nurse Partnership (FNP), based on work undertaken in the USA [20] was rolled out across the UK, with minimal evidence of benefit in a UK population. A large RCT (Building Blocks Trial) of 3251 women in England of the FNP programme found no positive association with the anticipated benefits [21]. This emphasises the need to ensure that interventions aimed at improving health outcomes are based on robust evidence of effectiveness and appropriate research is undertaken, with appropriate shorter and longer term follow-up to ensure that the intervention is effective in achieving the desired outcomes in the target population, prior to full implementation.

The current study was conducted as part of the New Dad Study (NEST), investigating whether it was feasible to use the Promotional Guide system to support first-time fathers’ mental health and wellbeing. This paper focuses on first-time fathers’ level of engagement with and acceptability of the intervention and reported impact on their mental health and wellbeing. Health visitors’ acceptability and experience of delivering this intervention was also undertaken but will be reported separately.

### Aims & objectives

The aims of this feasibility study were to:

1. Assess recruitment of first-time fathers, time to complete recruitment, and retention rates
2. Identify outcome measures relating to general health, mental health, couple relationship and social support which could be used in a future definitive study
3. Examine the potential changes following the use of the Promotional Guide system on first-time fathers’ mental health and wellbeing, as assessed at two to three months after their child’s birth
4. Explore whether first-time fathers would engage with the Promotional Guide system at planned antenatal and postnatal contact points and if they found it acceptable to be asked about their mental health and wellbeing
5. Obtain feedback from fathers about their experience of the intervention and the research process

## Method

A prospective observational cohort study was conducted incorporating quantitative and qualitative data collection methods. Feasibility and acceptability were assessed using a range of outcomes including recruitment and retention rates, data completeness, fidelity to study protocol, sustainability and adoption [22]. Relevant data were collated from study questionnaires and qualitative interviews.

### Study setting

This study was undertaken in four London administrative districts (known as boroughs in the UK), two of which were inner London and two outer London cities. The health visiting services for these settings were served by two NHS organisations (sites). Each site serves diverse socio-economic and cultural populations, with minority ethnic groups representing 44–69% of the overall total population of the boroughs selected [23]. These sites were chosen because the health visiting services used the Promotional Guide system as part of their Universal offer to all families.

### Recruitment

Expectant first-time fathers who accompanied their partners were recruited from antenatal clinics and health visitor contacts, through distribution of leaflets and posters which advertised the study website (www.newdadstudy.com). Research posters were displayed in antenatal clinics and ultrasound scanning departments of the two NHS trusts. Research midwives were asked to discuss the study with potential participants, and with the father’s permission, the names of those who were interested were forwarded to the researcher.

Fathers interested in participating were contacted by the lead researcher by telephone, and study procedures explained in detail and questions potential participants may have had about the study answered. Those who wished to participate were offered a participation information sheet, with details on how to complete study questionnaires sent via email. Postal questionnaires were available for anyone requiring them. Separate, written consent was not necessary as completing the questionnaires implied consent to participate. Participants were asked in the questionnaires whether they would be willing to take part in a one-off interview with the researcher. A subgroup of fathers who were willing were then invited to take part in individual qualitative interviews.

#### Inclusion and exclusion criteria

Biological and non-biological expectant first-time fathers, living within the health catchment area were included. Fathers were excluded if they:

- were non-English speaking, and not able to read or write in English
- experienced bereavement following neonatal death, stillbirth, pregnancy loss, sudden infant death
- had severe mental illnesses, such as schizophrenia, schizoaffective disorder, severe personality disorders, major depression and bipolar disorder.

English fluency requirements were applied for several reasons:

- There are over 300 different languages spoken across the four London boroughs included in this study and it was not practical to include all.
- Difficulties associated with using interpreters for qualitative interviews may mean that the essence of the interview may get lost during translation
- Fathers who did not speak English may have had specific needs relating to isolation and non-integration
- Although the Promotional Guides have been translated into Spanish and Japanese, the intervention was offered universally across the country in English only. Spanish and Japanese were not commonly spoken languages in the research sites.
- It was not practical to have all relevant documentation relating to this research translated to other commonly spoken languages due to resource and time constraints.

#### Sample size

As a feasibility study, the sample size was not powered to detect statistically significant differences in outcomes of interest. The aim was to recruit up to 50 participants, 25 from each NHS site. Teare et al. [24] recommended that an external pilot study to estimate key parameters for a definitive trial has at least 70 measured subjects (35 per group) in order to estimate the standard deviation for a continuous outcome. This suggested that 35 participants would be sufficient for this type of cohort study. To allow for drop-out and to enable more reliable estimates of change in the outcome measures, up to 50 expectant fathers were to be recruited across the two sites.

Qualitative interviews were planned with up to 15–20 participants, to enable data saturation to be reached, with no new information forthcoming [25].

### Data collection

Data were collected from fathers through online pre and post intervention questionnaires, and semi-structured telephone interviews.

#### Quantitative data

Participants were asked to complete the baseline questionnaire over a period of seven months (June – December 2018). Fathers were emailed a link to the web-based questionnaire on the Survey Monkey platform (created specifically for this study) which included questions on their socio-demographics and study outcome measures, between 24 and 28 weeks of their partner’s pregnancy and prior to the antenatal Promotional Guide contact with the health visitor (between 28 and 32 weeks of partner’s pregnancy). Following completion of the antenatal (baseline) questionnaire, participants were emailed by the researcher acknowledging their participation and informing them that a second questionnaire would need to be completed around two to three months following their baby’s birth, for which a reminder would be sent. Two months following the birth, the participants were sent another email with a second web link for the postnatal questionnaire and instructions for completion, along with the participation information sheet. The postnatal questionnaire contained the same outcome measures as in the antenatal questionnaire, and additional questions relating to Promotional Guides and men’s experience of health visiting services in the antenatal and postnatal period. As the postnatal Promotional Guide contact is typically delivered around 4–8 weeks after the birth of the baby, the postnatal questionnaire was completed after this period. If participants did not respond to the initial request, email and text reminders were sent at one-to-two-week intervals, with a maximum of three reminders sent. None of the participants required a postal questionnaire to be sent, as all had access to the online version. The postnatal questionnaires were completed between January and June 2019.

#### Outcome measures

Three validated psychological health measures were included in both questionnaires along with validated measures of general health, couple relationship and perceived social support, due to their alignment to the concepts identified in the theory of change and logic model developed for the Promotional Guide system (Figs. 1 & 2).

- ***Short Warwick-Edinburgh Mental Well-Being Scale (SWEMWBS)*** [26] includes a broad concept of positive mental well-being and incorporates both eudaimonic and hedonic perspectives on well-being [27]. The scale has been widely used in the UK, including in the Health Survey for England in 2016.
- ***Edinburgh Postnatal Depression Scale (EPDS)*** [28, 29] measures parental depression in the antenatal and postnatal period, and is validated for use with English speaking fathers [30]. It has been used in previous studies of fathers’ mental health in the perinatal period [31–35].
- ***General Anxiety Disorder 7-item Scale (GAD7)*** [36] is a self-reported questionnaire for screening and measuring severity of the four most common anxiety disorders (Generalised Anxiety Disorder (GAD), Panic Disorder, Social Phobia and Post Traumatic Stress Disorder).
- **EQ-5 L-5D** [37] is a commonly used standardised instrument for generic measure of health status internationally developed by the EuroQol Group. The version used has been validated in diverse patient populations [38].
- ***Couple Satisfaction Index (CSI)*** [39]: As paternal mental health is interlinked with wider aspects of fatherhood such as couple relational functioning, this scale enabled the assessment of fathers’ satisfaction with their relationship during their transition to fatherhood.
- ***Multidimensional Scale of Perceived Social Support (MSPSS)*** [40]: Lack of adequate social support has been linked to depression in fathers during the perinatal period [32, 40, 41]. This scale was selected due to its good internal reliability as a scale overall and for each subscale [40].

#### Qualitative data

The questionnaires included open-ended questions relating to father’s health and wellbeing, experience of fatherhood and perinatal mental health. Fathers were also asked to describe any additional support they would have found helpful during their transition to fatherhood.

At the end of each questionnaire participants were asked to indicate whether they were happy to be contacted by the researcher to take part in a brief interview to talk about their experiences. Twenty-nine (64%) of the 45 men stated ‘yes’. A sub-group of 17 of these fathers were invited to participate in in-depth qualitative interviews following completion of the second questionnaire. The plan was to interview men from three categories: those not involved in the Promotional Guide contacts, those fully involved in the Promotional Guide contacts (i.e. participated both antenatally and postnatally), and those who only received one contact. However, as none were fully involved with the intervention, a combination of those not involved with the Promotional Guide contacts and those partially involved were invited to participate in the interviews. Of the 17 men invited 10 responded. Initially it was planned to recruit 15–20 fathers, however after 10 interviews, no new information was forthcoming and data saturation was deemed to have been achieved. If saturation had not been reached, then the remaining men would have been invited to participate. The interviews were conducted using an interview topic guide to enable better understanding of the processes and underlying mechanisms in relation to context, setting, professionals and patients (Appendix – A) [42]. All fathers chose to participate in telephone interviews (rather than face-to-face interviews), with most interviews conducted during evenings and weekends. Participant information sheets were provided, and written consent obtained via email prior to each interview.

### Data analysis

#### Quantitative data

Data informed the main feasibility study outcomes relating to recruitment uptake, intervention participation, and follow-up rates. Mean and standard deviations were calculated for variables which were approximately normally distributed, and medians and inter-quartile ranges calculated for those not normally distributed. Individual characteristics that were categorical (e.g. religion, ethnicity) were described using frequencies and percentages. Mean and standard deviation estimates for pre-post change in SWEMWBS, EPDS, and GAD7 were derived as these could be used to inform sample size calculations for future larger study.

The statistical analyses were performed using IBM SPSS version 25.

#### Qualitative data

Each interview was transcribed using an external approved transcription company, with the principle of anonymity in mind and a confidentiality agreement in place. Data were analysed using framework analysis and the five steps of data management for thematic analysis [43] to include familiarisation; constructing an initial thematic framework; indexing and sorting; reviewing data extracts; and data summary and display. Framework analysis was chosen over other qualitative approaches due to its ability to answer specific research questions [44], in this case questions relating to the use of Promotional Guides with fathers in practice. It allowed the categories and themes identified in the data from the questionnaires to be explicitly and systematically considered, while also facilitating enough flexibility to detect and characterise new themes that emerged from the interview data [45]. NVivo (version 11) was used to facilitate this process.

### Patient and public involvement and engagement (PPIE)

A PPIE group of four first-time fathers provided expert advice to all project stages, including study design, data collection instruments, recruitment strategy, and study logo. In addition, health visitors and midwives provided feedback and advice throughout this project, with external experts providing additional support.^1^

## Results

### Recruitment and retention rates

In total, 86 fathers were interested in participating, the majority (*n* = 78) referred by Research Midwives at the study sites. Eight fathers contacted the researcher directly. Seven men did not meet inclusion criteria and were excluded and 79 were invited to complete the baseline questionnaire, 52 (66%) of whom did so.

**Fig. 3:**
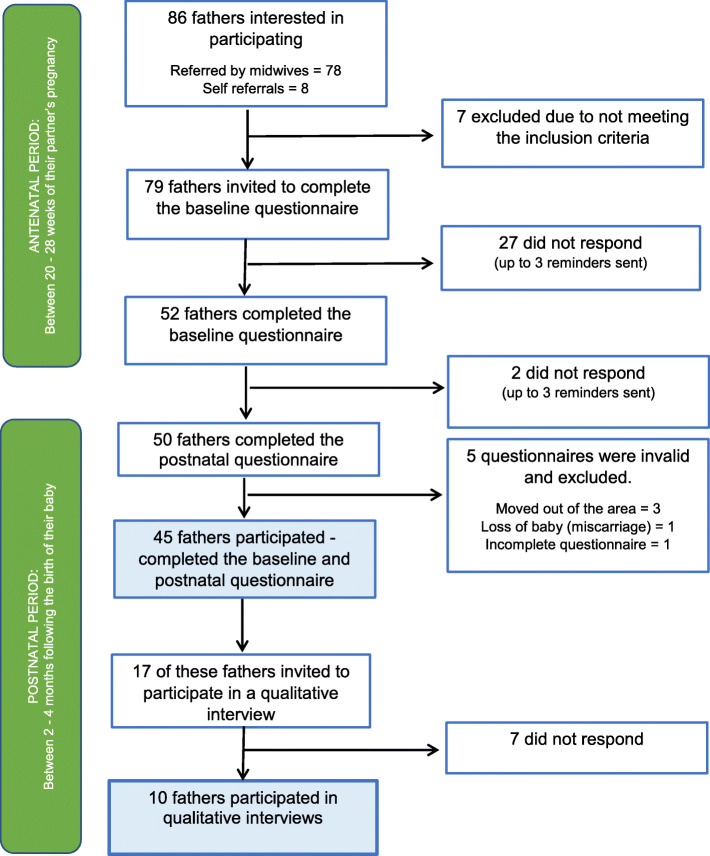
Flow diagram of the recruitment process

Of the 52 men, 50 also completed the postnatal questionnaire, a follow up and retention rate of 96%. Five of the 50 questionnaires however were invalid (three participants had moved out of the area, one lost their baby due to a miscarriage, and one questionnaire was incomplete). Data presented are based on the 45 men who completed baseline and postnatal questionnaires (Fig. 3).

### Participant characteristics

The majority of participants (*n* = 32) were aged between 30 and 39 years (71%), seven (16%) aged between 25 and 29 years and six (13%) 40–44 years. Nineteen men (42%) were White British; eleven (24%) White other; seven (16%) Indian; three (7%) Asian; three (7%) Mixed ethnic group; one (2%) Black African; and one (2%) identified as ‘other ethnic group’. For 29% (*n* = 13) of these men, English was not their first language, but they completed the questionnaires and interviews in English. Most (91%, *n* = 41) were either in full-time employment or self-employed, with 9% (*n* = 4) reporting to be in part-time employment. Annual income ranged from just over £5000 to over £61,000. Of these, only one man reported to earn under £15,000 per year. Education levels ranged from GCSE (high school certificate) to doctorate, with 53% being educated up to degree (or equivalent) level. All 45 men were in a couple relationship with their baby’s mother and of these 30 (67%) were married. Only one father did not co-habit with his partner and baby at the time of the study. Full participant details are presented in Table 1.

**Table 1:**
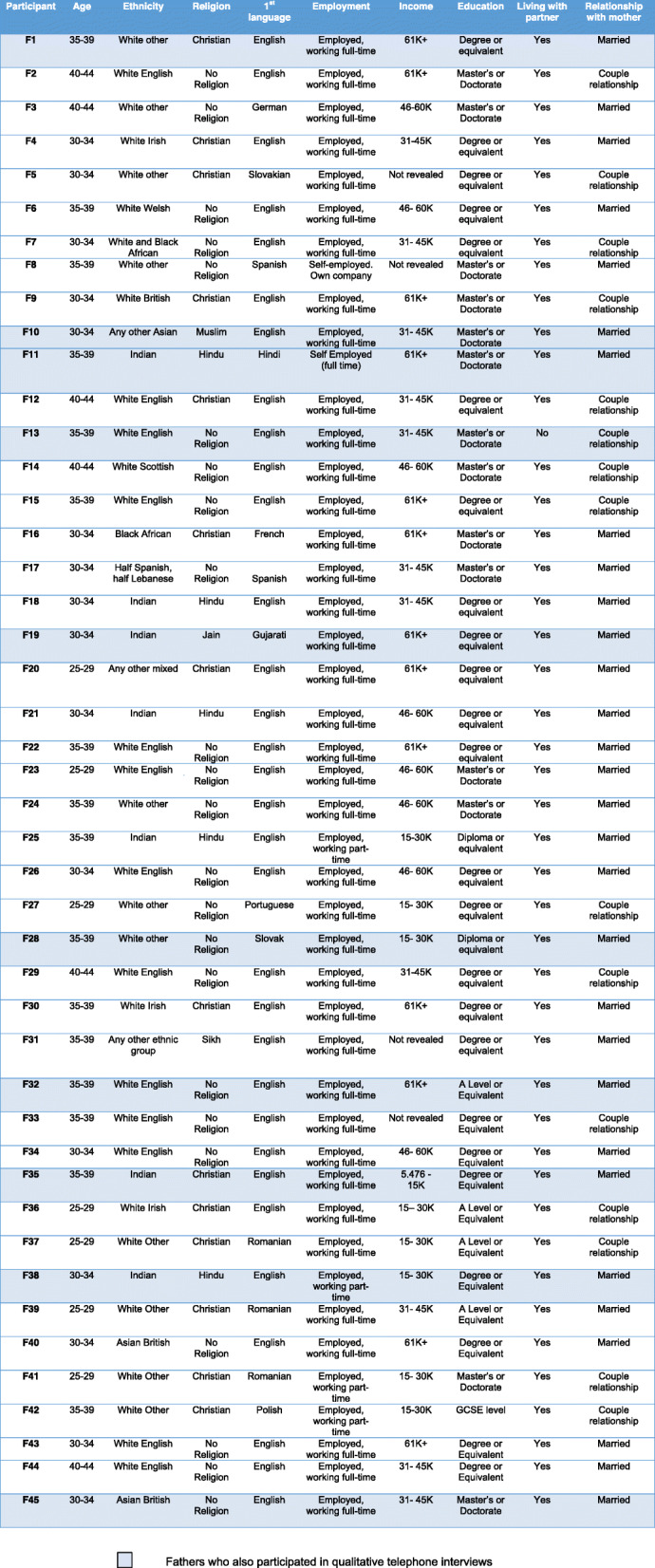
Characteristics of first-time father participants

### Feasibility of collecting outcome measures and impact

Questionnaire measure completion was high. Mean and standard deviation were calculated for all outcomes. Median and inter-quartile ranges were also calculated for EPDS, GAD7, CSI and MSPSS (Table 2).

**Table 2:**
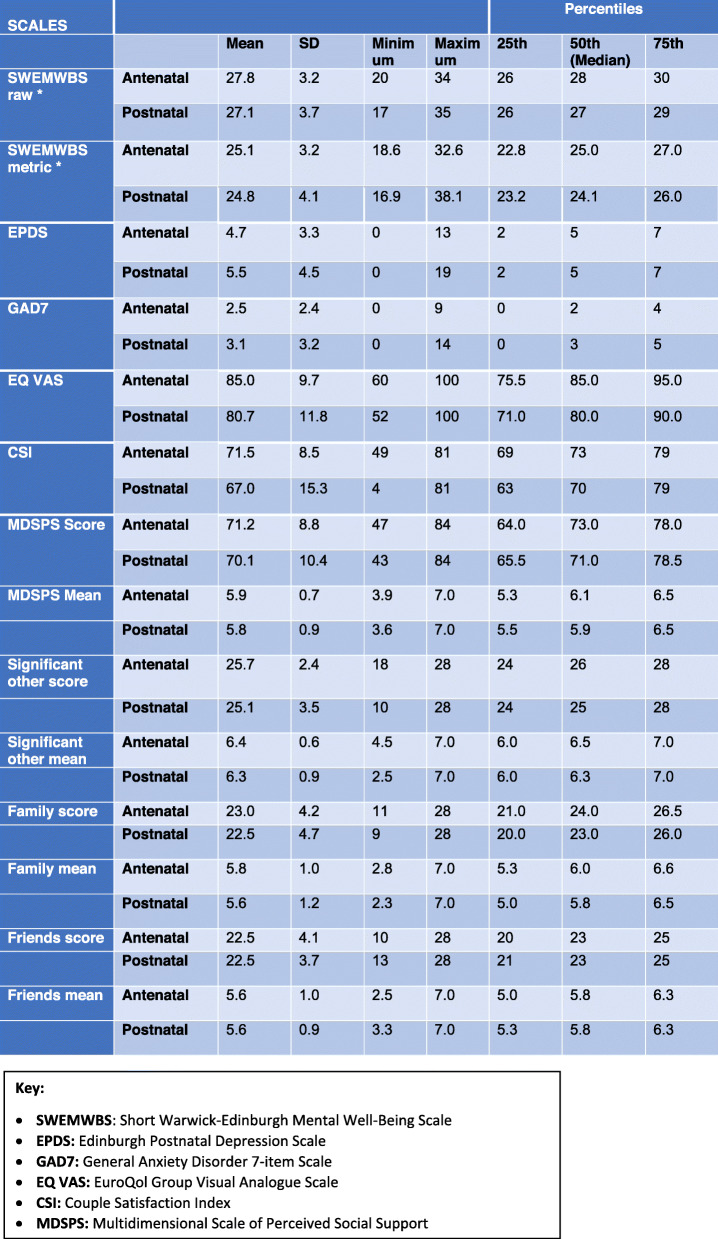
Summary of outcome measures in antenatal and postnatal questionnaires

*For SWEMWBS, the raw scores were transformed to metric scores using the standardised conversion table provided by the scale developer

#### Mental wellbeing using SWEMWBS

The cut off scores used were based on those used in an evaluation to establish national norms for mental wellbeing based on the 2010–2013 Health Survey data for England [46]. A cut-off point of 28 and above was considered as high mental wellbeing indicating positive mental health, 20–27 as average, and below 20 as low mental wellbeing [46]. The mean (SD) metric scores for first-time fathers’ mental health and wellbeing at both time points [Antenatal = 25.1 (3.2), postnatal =24.8 (4.1)] suggested participants had ‘average’ mental wellbeing, similar to the English populations norms for men using SWEMWBS, the mean (SD) being 23.7 (3.92) [46]. The minimum metric score in the antenatal period was 18.6, with only one man reporting low mental wellbeing (score < 20). In the postnatal period the minimum metric score was 16.9, with seven men reporting low mental wellbeing (scores = 19.98, 19.98, 16.88, 18.59, 19.25, 19.25, 19.98). The maximum metric score antenatally was 32.6, with nine men reporting high mental wellbeing (score ≥ 28); in the postnatal period the maximum metric score was 38.1, with seven men reporting a score ≥ 28.

#### Depression using EPDS

The mean (SD) score was 4.7 (3.3) in the antenatal period, and 5.5 (4.5) postnatally. The highest score in the antenatal period was 13 and in the postnatal period 19, the median for both time points being 5.

The cut-off point used to indicate possible depression was an EPDS score of ≥10, with 12 or more suggesting major depression [47–49]. Of the 45 men, 18% (*n* = 8) reported a score of ≥10 on at least one point in time during the perinatal period, with 13% (*n* = 6) reporting a score of 12 or more. Seven of these men had higher scores postnatally, suggesting depressive symptoms potentially increased. Two men had an EPDS score of 13 antenatally, with one increasing further in the postnatal period to 15, and the other reducing below the cut-off point (EPDS score = 7).

#### Anxiety using GAD7

The cut-off point used was a score of 10, with scores of 10–15 suggesting moderate anxiety and over 15 severe anxiety [35]. The mean (SD) GAD-7 score was 2.5 (2.4) antenatally, increasing to 3.1 (3.2) postnatally. Overall, there was a negative shift in the postnatal period with the median score increasing from 2 to 3. The maximum score [9] in the antenatal period remained below the cut-off point but increased to 14 in the postnatal period. Two men (4%) reported a score of over 10 (individual scores of 14 and 12), suggesting moderate anxiety. Both also scored high on the EPDS (19 and 14 respectively) and low on the SWEMWBS (16.9 and 18.6 respectively) in the postnatal period.

#### General Health using EQ-5 L-5D

There were no changes in ‘self-care’ functions at baseline or postnatally (Table 3). For usual activity there were six shifts in the negative direction. Forty-three (96%) men reported no problems with carrying on with their usual activity in the antenatal period and two (4%) reported slight problems. Postnatally 37 (82%) reported no problems, seven (16%) slight problems and one (2%) moderate problems with doing their usual activities.

**Table 3:** Distribution of EQ-5D-5L dimension responses at baseline and postnatal period

Dimension	Antenatal n (%)	Postnatal n (%)
**Mobility**		
No Problem	44 (98%)	43 (96%)
Slight problems	1 (2%)	2 (4%)
**Self Care**		
No Problem	45 (100%)	45 (100%)
**Usual Activity**		
No Problem	43 (96%)	37 (82%)
Slight problems	2 (4%)	7 (16%)
Moderate problems	0 (0%)	1 (2%)
**Pain/ Discomfort**		
No Problem	41 (91%)	39 (87%)
Slight problems	4 (9%)	6 (13%)
**Anxiety/ Depression**		
Not anxious/ depressed	37 (82%)	31 (69%)
Slightly anxious/ depressed	5 (11%)	13 (29%)
Moderately anxious/ depressed	3 (7%)	1 (2%)

For anxiety and depression, there were eight shifts in the negative direction. Antenatally, 37 (82%) men reported they were not anxious or depressed, five (11%) were slightly anxious or depressed, and three (7%) moderately anxious or depressed. Postnatally, 31 (69%) reported no anxiety or depression, 13 (29%) slight anxiety or depression, and one (2%) moderate anxiety or depression. Three men who reported anxiety and depression on this scale also scored high on the EPDS or on both the EPDS and GAD-7.

The EQ-5D-5L scale also includes the EQ VAS, a visual analogue scale (ranging from 0 to 100) to record the respondent’s self-rated health. The mean (SD) EQ VAS was 85 (9.7) in antenatal period and 80.7 (11.8) in the postnatal period; and the median (IQR) as 75.5 (85–95) in the antenatal period and 71 (80–90) in the postnatal period, suggesting a slight decline over the two time points.

#### Couple satisfaction using CSI

CSI scores can range from 0 to 81, with higher scores indicating higher levels of relationship satisfaction. CSI-16 scores falling below 51.5 suggest notable relationship dissatisfaction. The CSI mean (SD) was reported as 71.5 (8.5) antenatally and 67 (15.3) postnatally. There was one outlier, whose postnatal score was only 4, a marked decline from their antenatal CSI score of 50. This participant also scored high on the EPDS and GAD-7 scales, reported anxiety and depression on the EQ-5D-5L, reported low mental wellbeing on the SWEMWBS and his EQ VAS scores reduced from 85 to 70 in the postnatal period.

#### Perceived social support using MSPSS

This scale is divided into factor groups relating to source of the social support, namely family, friends and significant other. The overall score ranges from 12 to 84, higher scores indicating higher levels of perceived social support. Mean scores of 1 to 2.9 suggest low support; 3 to 5 moderate support; and 5.1 to 7 high levels of perceived social support.

The overall MSPSS score antenatally was 71.2, which decreased to 70.1 postnatally. The mean (SD) overall score was 5.9 (0.7) antenatally and 5.8 (0.9) postnatally, suggesting high levels of social support in both time points. This finding was consistent across the separate subscales, with the mean (SD) score for ‘significant other’ being 6.4 (0.6) antenatally and 6.3 (0.9) postnatally; for ‘Family’ being 5.8 (1.0) and 5.6 (1.2); and for ‘Friends’ being 5.6 (1.0) and 5.6 (0.9) respectively. Although the lowest overall mean score for MSPSS was 3.9 antenatally and 3.6 postnatally suggesting moderate levels of support, the minimum mean scores for two of the subgroups showed lower levels of support postnatally compared to the antenatal period (the minimum mean score for ‘significant other’ dropped from 4.5 to 2.5; and for ‘family’ from 2.8 to 2.3). The lowest mean score for ‘friends’ however increased from 2.5 in the antenatal period to 3.3 in the postnatal (Table 2).

While antenatal and postnatal outcome measures were collected from 45 participants, none reported receiving the intervention at both time points (full intervention). Seven (16%) participants reported receiving the intervention at only one point in time (Antenatal Promotional Guide *n* = 3, Postnatal Promotional Guide *n* = 4). Of these seven, one father was also interviewed (discussed later) where it transpired that he had not actually received the intervention. Due to the very small number of fathers receiving the intervention (less than 14%), further analysis of differences between the two groups (intervention vs usual care) was not carried out. The results presented in this section therefore reports the ‘current states’ for fathers in the antenatal and postnatal period and the changes between the two timepoints rather than any changes associated with the Promotional Guide intervention.

### Feedback from fathers – experience, engagement, mental health and research process

Twenty-nine (out of 45) men had indicated that they were happy to be contacted for the interview (64%). Ten men were interviewed, some of whom had no involvement with the Promotional Guide contacts and some who had. The demographic details of the men interviewed are shown in Table 1.

Data were analysed using Framework analysis. Six major categories were identified:

1. Experience of health visitor contact
2. Experience of Promotional Guides
3. Experience of perinatal health services
4. Experience of fatherhood
5. Fathers’ mental health and wellbeing
6. Experience of the research process

### Experience of health visitor contact

#### Invitation to attend

##### Feedback from questionnaires

Men were asked if they were invited to attend a planned appointment with the health visitor when their partner was 28–32 weeks pregnant (the appointment at which Antenatal Promotional Guide is used), 11 (24%) of whom stated ‘yes’. Thirteen (29%) men had attended this appointment, which included all 11 who were invited and two who were not. Similarly, men were also asked whether they were invited to attend a planned appointment with the health visitor when their baby was around 6–8 weeks old. Over half reported being invited (*n* = 25, 56%), 17 of whom attended and eight did not (five due to work commitments, three did not specify). In addition, five men who were not invited also attended, making it 22 (49%) in total.

##### Feedback from interviews

None of the 10 fathers were explicitly invited to attend any appointments with the health visitor. As one father stated “*… it was never an explicit appointment for me”* (F11). Some were present during home visits in the postnatal period, as one father explained *“they didn’t specifically ask me to be at home when they came, so they didn’t have to ask me specifically, I was present, so they didn’t have to invite me or anything, I was just there in the same room with P [wife] and the baby*” (F19).

#### Visits from the Health visitor

No participant recalled receiving a visit from the health visitor in the antenatal period, as one father said “*I don’t think we had any health visitors prior to her giving birth*” (F38), while another said “*nobody came to the house when she* [wife] *was pregnant*” (F35).

One father talked about seeing different health visitors in the postnatal period meaning there was no opportunity to build a relationship with any single practitioner: “*there were different ones that came and so there wasn’t really a relationship as such*” (F13).

#### Involvement during health visitor contacts

Men’s experiences with the health visiting service varied. Some felt very involved during the consultations (postnatal) and described feeling “*very much part of the conversation*”, One father said “*I was being listened to, they were asking me specific questions as well. Not just about me but about how I was perceiving my wife’s state of mind or physical exhaustion to be*” (F19). Another felt the health visitor was “*trying to involve both parents, asking different types of questions, observing the behaviour, how we* [they] *talked to each other …*. *I think it was like a 50/50, based on* [the parents’ needs]” (F28).

Some described feeling “*not really that involved, …. when the health visitor came it was sort of talking to L* [partner] *but I was sort of sat on the sofa as well, and she didn’t really sort of engage with me really*”. This father accepted not being spoken to because he justified that his partner “*was the one who was pregnant and I* [he] *sort of felt as if I* [he] *was sort of the support person*” (F13).

Some fathers described health visitors as task oriented where “*they came round, weighed, measured, checked over, asked if we had any questions and then kind of said goodbye*” (F32). “*It was never them saying to me, “Do you have anything to say, would you like to know anything?””* (F35).

### Experience of promotional guides

#### Feedback from questionnaires

Three fathers reported the Promotional Guide being used antenatally and four postnatally. One man recalled only one topic card was discussed, ‘Our labour & our baby’s birth’, while another stated that ten of the eleven topic cards were discussed. One father who originally indicated in the questionnaire that all eleven topic cards were discussed during the antenatal visit (F32) could not recall seeing any topic card or the Promotional Guides when asked during his interview.

Four men reported the Promotional Guide topic cards being discussed in a postnatal visit, including ‘Our emotional wellbeing’, ‘Becoming a mum, a dad & parents’, ‘Our baby’s development’; ‘Caring for our baby’, ‘Our baby’s cues’.

#### Feedback from interviews

When asked about the use of the Promotional Guides, fathers’ responses included “*that doesn’t ring a bell*” (F19); “*no, I can’t remember that being the case*” (F13); “*I don’t recall that happening*” (F10); “*no, absolutely not, no. So, the first time I heard of that was through your study which was quite recent actually*” (F38); “*no, there was nothing of that sort*” (F45). This was despite giving fathers an explanation of what the Promotional Guides were and what the topic cards may have contained.

### Experience of perinatal health services

#### Feedback from questionnaires

The fathers described positive and negative experiences. One father was “*very impressed from start to finish, the care at the hospital during labour was incredible, follow-up midwife appointments were good, the health visitor provided lots of info …*” (F2).

Despite this, many did not feel included or involved by health professionals. One father described the postnatal ward as feeling “*a little hostile to fathers at times*” (F6). Others stated how the services were mainly geared towards the mother (F15, F25, F28, F36, F45), with one father describing his experience as “*being a passenger rather than participant*” during the perinatal period (F24).

The support in the postnatal period was “*less thorough*” with “*no immediate continuous support with postnatal issues comparable to the prenatal service*” (F3). The lack of adequate communication between health professional was also highlighted (F1). In addition, fathers not being acknowledged by health professionals featured strongly in the feedback (F2, F26, F29, F34, F35, F36, F45).

#### Feedback from interviews

Some fathers had positive experiences of health professionals in the postnatal period, for example “*we were massively, massively impressed by the care and support we received from the NHS and especially the … .hospital staff, they were exceptionally helpful for my wife*” (F19). Fathers talked about feeling grateful for the services they received, in particular appreciating the home visits by the midwives following their baby’s birth (F1). One father also acknowledged that “*you guys* [health professionals] *do as much as you can*” (F38).

In contrast, others reported a lack of support, particularly in the postnatal period. One father of a seven-month-old felt that support following the birth was non-existent both for himself and his partner “*I’d say in terms of … initial dad support, … there hasn’t been anything but since then there hasn’t been anything at all for me.. … ..so it’s that lack of support just continues*...” (F13).

Fathers who attended antenatal classes had to use their own initiative, as this father stated “*I think there is not much ongoing for fathers, I would feel, unless you really want to get involved. And you seek the information, you seek advice*” (F28). Inpatient postnatal care was described by one father as being *“… a bit different, things felt like they were a bit disorganised, unorganised*” (F45). This father also felt responsible and unsupported when his partner and baby were separated for over an hour after birth.

### Experience of fatherhood

#### Feedback from questionnaires

Many fathers described feeling tired and sleep deprived, which increased their stress levels and anxiety in the postnatal period (F2, F15, F18, F24, F34, F36, F42). One father was “*occasionally snappy, angry and impatient …”* (F26). Another stated that due to being sleep deprived, his mood could “*fluctuate quickly*” (F29).

Financial responsibility was also a concern, “*more concerned about long term finances since becoming a father*” (F20); “*it’s too stressful, I’m always tired, have to work hard for money, its expensive*” (F37).

They wanted to spend more time with their child (F45), and the 2 week’s paternity leave was not long enough (F19, F32).

Some men were concerned about their own weight gain in the postnatal period; “*I feel a bit fat* (F45); “*I have spent almost zero time doing any exercise, I’ve gained weight*” (F1); and “*would like to be in better shape and go back to doing some exercise*” (F17).

#### Feedback from interviews

Fathers found it difficult to go back to work and be separated from their baby. According to one father “*for that first six months it’s almost harder for the father because, you know, I have to go to work and so I see him for, you know, 30 minutes in the morning and then I get back and I see him for an hour in the evening, and you’ve got a son and where you’ve got to relate that to living to the weekends. And so I’d say in a way it’s the type of support that is required is slightly different for that because it’s almost sort of dealing with separation from your son and it’s something which is quite difficult*” (F13).

Breastfeeding difficulties and a lack of support to overcome these were challenging. One father’s wife felt ‘judged’ because she was not able to breastfeed “*There was something … an inadequacy with her rather than the other problems. So in the end we just went to a private lactation consultant to help us out because we tried multiple support groups, and everyone had such a different opinion. It was not scientific, it was more an anecdotal kind of set up*” (F11).

Increased anxiety was also mentioned in the postnatal period because “*if you are a father to a new born child and you have some kind of financial problems, the level of anxiety would definitely go up because you now have to worry about your children as well on top whatever your existing set up was”* (F11).

### Fathers’ mental health and wellbeing

### Enquiry by health visitors

#### Feedback from questionnaires

Only one of the 45 men was asked about his experiences or needs relating to becoming a father antenatally. Comments included “*it* [the antenatal contact] *was all about my partner and the baby*” (F36) and the father “*wasn’t asked anything beyond how I was doing*” (F19). Postnatally, only two fathers were asked about their own experiences or needs. Comments included “*the visit is focussed on the mother and the baby, the father does not appear to be on top of the list in terms of priority*” (F11).

#### Feedback from interviews

Most fathers were not asked about their own mental health and wellbeing by the health visitor during the perinatal period, as the focus was on the women: “*about my partner’s wellbeing? Very much so. Not so much my own*” (F10). Another said, “*they never asked anything to the birthing partner or the father, so they never ask are you feeling exhausted, are you feeling [over] … and are you okay?*” (F11).

Those asked about their own wellbeing were asked questions such as “*how I* [he] *was managing work and the baby and everything else*” (F19), or “*along the lines of, “How are you coping? Everything going okay? Are you getting much sleep?” so probably more in a soft way*” (F32) rather than being asked direct questions about mental health.

Health professionals were perceived as speaking to fathers in a more ‘*light-hearted’* way. According to one father “*it was mainly just kind of, you know, the odd jokes, you know, joke around as if it was my job to change the nappies, or, you know, look after … I have to look after my wife and the baby and sort of thing. So, I don’t have any sort of recollection of staff or health professional’s kind of taking my health into consideration*” (F45).

### Barriers to accessing support

#### Feedback from interviews

Fathers talked about barriers to accessing support for their own mental health and wellbeing and not being informed about antenatal or postnatal appointments with health professionals which were normally arranged directly with their partner. As this father described “*before the baby was born, I wasn’t really notified from my point of view, I think it was just my wife”* (F28).

Another barrier was the “*lack of visibility or lack of communication and, you know, when you go to the appointments at the hospital, there’s, you know, all of the literature and all of the stuff which is on the walls and is about more for the mother*” (F13).

Several men spoke about clinicians’ views on childbirth as a barrier to involving them; “*the main barrier in offering any kind of support to the fathers is the mindset that birth is all about the mother and the child, and everything else is a secondary consideration*” (F11).

### Need for better pre and post birth support

#### Feedback from questionnaires

Fathers identified several things that would support their wellbeing during their transition to fatherhood, including information about fathers’ groups, childcare and support services, “*feeding and general how-to-dos for caring for the baby*” (F19), tailored information for fathers, “*online videos and bitesize information*” (F40), and preparation for changes in new fatherhood. One father stated, “*Probably more that the health visitor shows interest in fatherhood and supports them too along with the mother*” (F35), while another wanted “*acknowledgment* [from health professionals] *that my life will also change*” (F45).

They wanted better facilities for fathers on postnatal wards so that they could better support their partners. As one father explained that there “*was just a bed over there and a very uncomfortable chair for me to be around with her and the baby, and given that I had not slept for more than 40, 45 hours, like it was quite physically exhausting to the extent that I literally slept on the floor*” (F11).

#### Feedback from interviews

Fathers wanted “*the ability to meet other people who are in the same situation*” (F13), through antenatal classes or groups, with adequate antenatal preparation considered important by most.

Similar to questionnaire responses, fathers wanted to be asked about their own wellbeing - “*some simple stuff. How are you feeling? You know, how are you doing? Do you have any concerns? But even maybe to build like a small little relationship every time with the father”* (F35). Some suggested a routine antenatal appointment for discussing the practical issues relating to new fatherhood, as well as to “*have a professional to talk to, to kind of just say how are you doing and, you know, any support, and, you know, just similar to what my wife had, mental health questions and all that sort of stuff*” (F45).

Offering the father a separate appointment to the mother was seen as being appropriate “*because if it is a man or a woman, if they are going through some sort of abusive phase, facing abuse rather, it would be easier for them to speak up separately*” (F19).

Fathers felt that they were not offered sufficient information about breastfeeding difficulties and in infant feeding classes *“it was almost presenting a utopian view of how feeding would come about, you know, you take the baby and you plonk him on it, and it just works like magic”* (F19).

### Experience of the research process

The fathers’ main motivation for taking part included: interest in the topic (F11, F45), being able to share their own views and experiences (F28), father’s mental health being an under-researched area (F10), contribute to research on fathers (F1, F11, F13, F19, F38, F45), and to benefit other fathers (F38).

This father summed up the views of most who participated: “*From my perspective, I feel like the fathers are sort of the forgotten entity when it comes to the pregnancy and the post pregnancy sort of thing. I wanted to be a part of contributing in any way that I could to make sure that this also an area of research or study that is taken up. Because more and more I see fathers being very, very involved in the child rearing, right from the very beginning and being supportive to their partners in their pregnancy … … I would say the gender roles are more fluid now, it’s not like the man is completely hands off, so I want to make sure that I can participate and contribute in any way that I can because I see that this is an evolution of the role for me*” (F19).

Fathers referred to several beneficial impacts of participating in the study, such as improving services for other fathers and involving fathers by asking their views. Completing the questionnaires allowed men to reflect on their own feelings about becoming a father, “*those questionnaires do make you think a lot actually about where you are as a person, and where you’re going as a dad, and how you’re feeling about things coming*” (F38). Participating in this study also enabled fathers to access additional resources which they may not have had accessed otherwise.

## Discussion

### Recruitment and retention rates

Historically, fathers have been underrepresented in health research [50–52]. The response rate for the current study was 66%, with a follow up and retention rate of 96%, with fathers included from diverse backgrounds. The recruitment and retention rates indicate that it is feasible to recruit first-time fathers into a study about their transition to parenthood.

### Feasibility of collecting outcome measures and impact

All men completed the outcome measures in both questionnaires suggesting it is feasible to use these measures in a future definitive study. None of the fathers however received the Promotional Guide intervention in its entirety as specified by the programme during the antenatal and postnatal period. Although seven fathers reported in the questionnaire that they received the intervention at one time point only, when one father was later interviewed, he reported that his health visitor did not actually use the Promotional Guide, meaning it is possible that the other six fathers also did not receive the intervention. This suggests the benefit of including qualitative interviews to provide a better understanding of health visitor contacts in a future evaluation. As the time period between the antenatal contact and fathers’ interviews was between four to 6 months, it is possible that the father who reported (in the questionnaire) to receive the intervention antenatally may have not been able to accurately recall its use. It is also possible that the health visitor did not explicitly explain the intervention, which may have resulted in him not being aware of it. Findings need to be considered in the design of any future study, and an additional interview after the antenatal contact considered.

While it is not possible to report on any potential changes following the use of the intervention in this study, the changes in the fathers’ ‘states’ in the antenatal and postnatal period provide useful information, contributing to the evidence on new fathers’ health and wellbeing during the perinatal period. The general health questionnaire (EQ-5D) suggested some deterioration in self-reported health postnatally compared to antenatally. A few fathers reported slight difficulty in carrying on with their usual activity prior to the birth, and more reporting slight or moderate problems eight to ten week postnatally. Mental wellbeing was reported as ‘low’ (on SWEMWBS) by one father antenatally, but by seven fathers postnatally. In this study 13% of men reported major depression (EPDS score > 12) during the perinatal period, higher than reported in recent meta-analyses studies [1, 53]. While most studies in the meta-analyses [1, 53] used self-reported questionnaires, some used structured or semi-structured interviews, and some used both methods. Assessment tools, timing of assessment and cut-off points also varied between studies, making direct comparisons difficult.

The EPDS scores in the current study were higher postnatally, similar to the anxiety scores, suggesting a correlation between low mental wellbeing, depression and anxiety. Use of different mental health assessment scales could provide a more comprehensive picture of first-time fathers’ mental health and wellbeing. Our previous studies explored how becoming a father could negatively impact on men’s mental health and wellbeing [54, 55]. Our systematic review reported on studies where fathers experienced increased levels of tiredness due to lack of sleep, often resulting in increased stress, frustration and anger in the postnatal period [54, 56–60]. These factors could explain the deterioration in general health, low levels of mental wellbeing, and the increased rate of depression and anxiety reported in the postnatal period by men in the current study.

The CSI results suggested a decline in couple relationships in the postnatal period, which could have been triggered by depression or conversely trigger symptoms of depression in parents [61], these findings also reflecting our systematic review [54]. Some men perceived that the social support they received from their ‘significant other’ was lower in the postnatal period, compared to the antenatal period which could reflect the deterioration in couple relationship following the birth. At the same time, some men perceived the support they received from friends increased postnatally, which could reflect support received from other new fathers or increased interaction with friends following the birth of their baby.

The theory of change and logic model (Figs. 1 & 2) outlined the mechanisms through which the use of Promotional Guides could improve mental health and wellbeing of first-time fathers. Mean and standard deviation estimates for pre-post change in SWEMWBS, EPDS, and GAD7 could be used to inform sample size calculations for a larger study as potential primary outcome measures. While there is some overlapping of questions in the EQ-5D, SWEMWBS, EPDS and GAD-7 scales, using them collectively could provide a more comprehensive picture of an individual’s mental health and wellbeing, while facilitating validation of data obtained through different instruments. All study outcome measures had previously been validated for use with men and were acceptable to the fathers.

### Feedback from fathers

No fathers could recollect the Promotional Guides or topic cards, but some commented on other information provided by health visitors including information on local services, health promotion advice and leaflets. Health visitors trained in the Promotional Guide system may have used the principles of the Family Partnership Model during their consultation without using the Promotional Guide material; however, this could not be ascertained.

In the questionnaires, three fathers reported the Promotional Guide being used during the antenatal appointment and four postnatally but it is clear that no fathers reported receiving the Promotional Guide intervention as planned. It is not possible to draw any conclusion from data collated about how the intervention was delivered and whether the Promotional Guides were used as a basis for the contact in the first place. This is a huge omission in the implementation of an intervention intended to be a universal offer for all families.

Men described increased tiredness, sleep deprivation, stress and anxiety and that their mood could fluctuate and result in them feeling “snappy, angry and impatient”, findings supporting our earlier work [54, 55]. New fathers worried about their increased financial responsibility and not being able to spend enough time with their child, again similar to previous findings [54, 55]. For some men, returning to work following paternity leave was hard, their 2 weeks statutory paternity leave insufficient to enable bonding with their baby. Fathers not wanting to ‘miss out’ on their child’s development and ‘feeling they were abandoning their partner and new child at a time when they were needed’ has been reported in other recent studies [55, 62]. Although commonplace for health visitors to prepare mothers for going back to work after having a baby [19], none of the fathers in this study were offered such support.

‘Shared Parental Leave’ (SPL) was introduced in the UK in 2015 [63], to help eligible parents to better combine work with family life. For eligible couples, it means women can cut their maternity-leave short (after the mandatory recovery period within maternity leave) and share the remainder of their entitlement (up to 50 weeks) with their partner as SPL [63]. Despite this, the uptake of SPL was only 1% in 2017/2018 [62]. SLP was not mentioned by any of the fathers we interviewed, and it is not known if whether they were aware of it or had even considered it.

Many fathers felt they had neglected their own health needs and were particularly concerned about their weight gain. While perinatal weight gain in women has been subject to research [64, 65], weight gain in fathers during this period is less recognised. Saxbe et al. [66] identified several mechanisms that may contribute to paternal weight gain, including sleep deprivation, lack of physical activity, and increased calorie diets; as well as hormonal mechanisms such as decreased testosterone and increased cortisol levels; and psychological mechanisms such as depression and stress. While these possible associations require robust investigation, weight gain in expectant and new fathers is a serious public health issue, which may have lifelong health implications for men and their families.

Fathers’ experiences of supporting breastfeeding were challenging due to inadequate support and conflicting advice received from health professionals. Providing fathers with appropriate information about breastfeeding, with planned, on-going support following the birth to ensure that they can better support their partners has been highlighted previously [54, 67–69]. If fathers can provide better support, breastfeeding is likely to be more successful and women are likely to feel more confident with breastfeeding [70].

No fathers interviewed were exclusively invited to attend the antenatal or postnatal appointment with the health visitor. Of the fathers who completed the questionnaires, all who were invited to the antenatal appointment and most invited to the postnatal appointment with the health visitor, had attended the contact. It has been previously documented that fathers are more likely to attend appointments if they are explicitly invited [55, 71]. Men who attended the antenatal appointment found several things helpful related to the preparation for fatherhood, including what to expect during labour and the early postnatal period, and who to contact if they needed help. The postnatal visit was useful for reassuring parents and providing practical support relating to caring for their baby. Some fathers however did not find the postnatal contact useful and described the health visitor as not being a ‘good listener’ or interested in the father’s own needs. This highlights the importance of health visitors having appropriate skills and attributes to work effectively with both parents [72].

Fathers felt involved when the health visitor treated them as equal partners in the consultation. When this did not happen, fathers felt ignored by the health visitor and not considered as a parent in their own right. These views were not dissimilar to those reported in previous studies where men described being perceived as ‘useless’ and feeling like ‘bit of a spare part’. [55, 73, 74] The lack of adequate communication between health professionals, and fathers not being acknowledged by health professionals were common themes also reported previously [54, 55].

Most were not asked about their own mental health and wellbeing by the health visitor at any contact but if they were asked, it was “in a soft” or “light-hearted way”. Baldwin et al. [55] suggested fathers wanted to be asked direct questions about mental health and wellbeing, and most were willing to speak to health professionals if they knew they were there to support them as well as the mother and baby. This has major implications for providing an equitable service to both parents. Exclusion from antenatal appointments, antenatal classes and antenatal literature, were identified as gaps in the preparation of fathers for fatherhood by Deave et al. [75] Thirteen years on, this remains [54, 55]. Health professionals not treating fathers with equal importance as mothers, or as equal partners during the perinatal period is a barrier, preventing them from feeling involved.

Similar to previously reported findings, men wanted access to tailored information for fathers relating to the changes and adjustments in the transition to fatherhood; caring for their babies; and access to fathers’ groups and support services [54, 55]. It was clear that men wanted support to be available for fathers throughout the perinatal period, some suggesting routine appointments for ‘fathers only’ or ‘father only support groups’, while others, a combination of joint appointments with their partner, communication via email or social media platforms, and open access to a professional helpline throughout the perinatal period. Better facilities in the postnatal ward was highlighted as a gap, which has also been identified in previous studies [55, 76].

### Strengths and limitations

There are several strengths. Firstly, the recruitment and retention rates indicate that it is feasible to recruit first-time fathers into a study about their transition to parenthood. The fathers recruited found being involved in the study was acceptable and they understood the aims of the study. The recruitment approaches used meant that first-time fathers from diverse backgrounds were recruited as planned and, in the time planned. Fathers found research processes acceptable, with high completion of all outcome measures. Findings also showed the value of using different scales for measuring different aspects of mental health and wellbeing in fathers.

Study limitations include not being able to report on use of the Promotional Guide system and its impact. Six fathers who reported receipt of the intervention at one point in time in response to the questionnaire did not volunteer to be interviewed so it was not possible to explore the extent to which the Guides were used, if it was acceptable to them, their willingness to engage with it or confirm if it had indeed been used.

Unless fathers are routinely invited and involved in the Promotional Guide contacts, it is not possible to ascertain whether it is likely to be an intervention that supports men’s mental health and wellbeing during their transition to fatherhood.

### Recommendations for practice

A consistent approach to the delivery of the Promotional Guide system is required, in addition to better evidence of effectiveness before scale-up of the intervention. Fathers should be explicitly invited to antenatal and postnatal appointments with the health visitor and informed that the appointment is for the father as well as the mother.

Health visitors should avoid making assumptions that men perceive perinatal health services to be for ‘women only’, as this could act as a barrier to involving fathers. Health visitors should also enquire about fathers’ mental health and wellbeing, and ask direct questions as they do with mothers. Men are then more likely to express their own needs and engage more. Health visitors could help new fathers prepare for the changes in fatherhood and provide information and support in areas including breastfeeding, fathers’ own physical and mental health, attachment and bonding with baby, and SPL rights and going back to work. Fathers should be provided with details of all available support and resources including local support groups, websites, and helplines by health professionals in the perinatal period. For this health professionals need to be aware of services available for fathers. Overall, this study highlighted the need for better service provision and support for fathers, especially in the postnatal period.

### Recommendations for research

Further research is needed to assess the effectiveness of the Promotional Guide system used by health visitors with mothers and fathers. For this to happen, the implementation gaps identified in this study need to be addressed first. Further research could be undertaken with fathers utilising the outcome measures from this study and evaluation methodology to include a control group to test the effectiveness of Promotional Guides in improving fathers’ mental health and wellbeing.

More fathers should be included in perinatal and child health research to address the current gaps. The research strategies used in this study could help inform the recruitment and retention of fathers from diverse backgrounds. There is also little known about fathers’ physical health and weight gain in perinatal period and its relationship with mental health and wellbeing during this period. This is an area that would benefit from further research.

## Conclusions

This study considered the feasibility of conducting a future large trial to determine the effectiveness of using the Promotional Guide system with first time fathers, assessed in four different boroughs of London. It included a nested process evaluation to consider the acceptability, feasibility and fidelity of programme delivery from the fathers’ perspectives.

Recruitment and retention of first-time fathers were positive aspects of this study and the findings suggest that the chosen outcome measures for general health, mental health and wellbeing, couple relationship and social support could be used with fathers in a future definitive study. There were a number of implementation gaps identified for the use of Promotional Guides with fathers that need to be addressed before a trial could be undertaken, and a pilot trial is recommended prior to a full scale trial [77]. The findings nonetheless provide an insight into men’s experiences of first-time fatherhood, their contacts with health professionals during the perinatal period and their experience of the research process. Recommendations from this study could inform better health practices to support fathers’ mental health and wellbeing during the perinatal period.

## Supplementary Information

**Additional file 1.**

## Data Availability

The datasets used and/or analysed during the current study are available from the corresponding author on reasonable request.

## Footnotes

1 Adrienne Burgess (Joint CEO and Head of Research) at the Fatherhood Institute and Dr Crispin Day (developer of the Promotional Guide System) from the Centre for Child and Parent Support.
