# Supplementary material for: A mixed-methods feasibility study of an intervention to improve men’s mental health and wellbeing during their transition to fatherhood
Source: BMC Public Health. 2021 Oct 8;21:1813. doi: 10.1186/s12889-021-11870-x (PMC8501623; doi:10.1186/s12889-021-11870-x)
Supplement: Supplementary file 1 — Additional file 1. [file 12889_2021_11870_MOESM1_ESM.docx]

**Appendix – A**

**Qualitative Interview Topic Guide for Fathers**

**
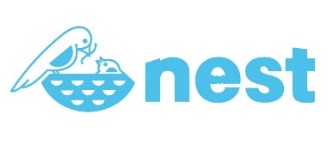
**

***The interview should take no longer than 60 minutes and you are free to leave the study at any point. Do you have any questions before we start the interview?***

1. The level of involvement with the intervention and the reasons for this.

- Not involved in the intervention: to ascertain reasons for non-involvement
- Fully involved in the intervention (both antenatally and postnatally): what helped them to fully engage
- Partially involved in the intervention (only antenatally or only postnatally): to determine reasons for partial participation and any barriers

1. Fathers’ perception of the intervention – Were you exclusively invited to take part in the Promotional Guide contacts – antenatally and postnatally with your partner? In what ways did you find the Guide material included you as a father? How were you made aware that the Promotional Guides are aimed at fathers as well as mothers?
2. As a father, to what extent were you given the chance to talk about your feelings and emotional well-being and any concerns you have? In what ways, did the Guide materials used by the practitioner whom you saw help you plan ways to help you with any emotional concerns that you had?
3. As a father, what aspects of the Guides did you find helpful? How would you describe the relationship between you and the practitioner?
4. How were the topics chosen for discussion during these visits? As a father, how would you describe your participation in this?
5. What changes did you make following the discussions taken place with the practitioner using these guides, if any? How did you follow up on any advice given during the Promotional Guide visits?
6. What were the barriers and facilitators influencing engagement of fathers with the Promotional Guide System?
7. What attracted you, as a father, to participate in the study?
8. What was it like to complete the questionnaires at 3 different points for this study?
9. Is there anything that could have been done better?

***I would like to ask you a few more questions about your antenatal and postnatal contact with the health visitor.***

**Fidelity Checklist:**

1. Did you feel listened to and heard by your practitioner?
2. Did you feel that your practitioner understood and appreciated you, your family and your circumstance?
3. Which of the following most closely reflects your practitioner’s use of the Promotional Guide materials and content during your contact?

- Your practitioner helped you to use the Promotional Guide Topic Cards as the basis of your contact
- Your practitioner helped you to use the Promotional Guide Topic Guide as the basis for your contact
- None of the above

1. Which of the following most closely reflects your practitioner’s use of the Family Map with you and your partner?

- Your practitioner helped you to use the Family Map as the basis of your contact
- Your practitioner helped you to explore some of the Family Map during your conversation
- Your practitioner helped you to make full use the Family Map to inform your conversation
- None of the above

1. Did your practitioner help you to identify specific main priorities?
2. Did your practitioner help you to make a written record your main priorities?
3. Did you make a written record of the main areas for improvement identified through your conversations with the practitioner?
4. Did your practitioner help you to make a written record of your shared goals and plan for achieving them?
5. Did your practitioner help you to identify specific family members, friends or other social supports to assist with your goal achievement and plans?

***This is the end of the interview. I would like to thank you very much for taking part in this study. Do you have any questions you would like to ask?***
